# Supplementary material for: The purine metabolite inosine monophosphate accelerates myelopoiesis and acute pancreatitis progression
Source: Commun Biol. 2022 Oct 12;5:1088. doi: 10.1038/s42003-022-04041-0 (PMC9556615; doi:10.1038/s42003-022-04041-0)
Supplement: Supplementary file 2 — Supplementary Information [file 42003_2022_4041_MOESM2_ESM.pdf]

Supplemental Materials

Purine metabolite Inosine monophosphate accelerates myelopoiesis and acute pancreatitis progression

Short title: inosine monophosphate on myelopoiesis and acute pancreatitis

Xiao-Min Luo[1,*], Sin Man Lam[2,*], Yuan Dong[1,*], Xiao-Juan Ma[3], Cen Yan[1], Yue-Jie Zhang[1], Yu Cao[1], Li Su[4], Guotao Lu[5], Jin-Kui Yang[6], Guanghou Shui[2,#], Ying-Mei Feng[1,#]

Affiliations:

1. Department of Science and Development, Beijing Youan hospital, Capital Medical University, Beijing 100069, China; 2. State Key Laboratory of Molecular Developmental Biology, Institute of Genetics and Developmental Biology, Chinese Academy of Sciences, Beijing 100101, China. 3. Center of Basic Medical Research, Institute of Medical Innovation and Research, Peking University Third Hospital, 49 North Garden Road, Haidian District, Beijing, 100191, China. 4. Neuroscience Research Institute, Peking University Center of Medical and Health Analysis, Peking University, Beijing 100191, China **5.** Pancreatic Center, Department of Gastroenterology, Affiliated Hospital of Yangzhou University, Yangzhou University, Yangzhou 225099, China. 6. Department of Endocrinology, Beijing Tongren Hospital, Capital Medical University, Beijing, China.

***Equal contribution to the study**

Correspondence to

Ying-Mei Feng MD, PhD, Email: yingmeif13@sina.com or yingmeif13@ccmu.edu.cn

Guanghou Shui PhD, Email: ghshui@genetics.ac.cn

Supplementary Table 1. Comparison of each metabolite in GMP cells of db/db mice at different ages.

Metabolites (nmol/10^6 cells)	GMP of 8-weeks-old mice	GMP of 24-weeks-old mice	P value
citric acid	11.27 (4.6)	13.57 (2.4)	0.52
isocitrate	0.036 (0.015)	0.038 (0.008)	0.87
alpha-ketoglutarate	1.34 (0.55)	1.31 (0.22)	0.87
succinate	0.95 (0.29)	1.56 (0.22)	0.063
fumarate	0.60 (0.18)	1.15 (0.29)	0.08
malate	0.10 (0.03)	0.15 (0.02)	0.07
oxaloacetate	0.07 (0.03)	0.07 (0.01)	0.75
lactate	0.94 (0.28)	1.99 (0.77)	0.30
pyruvate	0.75 (0.23)	1.01 (0.08)	0.07
fructose-6-phosphate	0.07 (0.03)	0.14 (0.02)	0.07
glucose-6-phosphate	0.13 (0.04)	0.24 (0.09)	0.008
fructose-1,6,-bisphosphate	0.29 (0.12)	0.54 (0.09)	0.08
uridine diphosphate-glucose	0.11 (0.04)	0.12 (0.02)	0.87
uridine diphosphate	4.97 (1.50)	7.56 (1.50)	0.77
erythrose-4-phosphate	0.67 (0.20)	1.24 (0.25)	0.18
ribose-5-phosphate	1.29 (0.39)	2.50 (0.47)	0.02
sedoheptulose-7-phosphate	0.58 (0.18)	1.22 (0.25)	0.005
adenosine monophosphate	0.62 (0.19)	0.77 (0.14)	0.55
adenosine diphosphate	0.24 (0.07)	0.33 (0.05)	0.52
adenosine triphosphate	2.42 (0.99)	3.50 (0.59)	0.08
guanosine diphosphate	0.17 (0.05)	0.56 (0.10)	0.08
guanosine triphosphate	0.08 (0.03)	0.18 (0.03)	0.02
inosine monophosphate	1.59 (0.50)	2.72 (0.43)	0.03

Data are expressed as mean (SEM). The statistical difference of each intracellular metabolite levels was analyzed by non-parametric Mann Whitney analysis. N=6-16 per group.

Supplementary Table 2. FACS antibody list.

antibodies	clones/catalog number	company
anti-mouse Sca-1 PerCP-Cy5.5, FITC	D7	eBioscience
anti-mouse Sca-1 PE-cy7	D7	BD
anti-mouse cKit PE	2B8	eBioscience
anti-mouse cKit APC-H7	2B8	BD
anti-mouse lineage cocktail APC	M1/70, 145-2C11, RB6-8C5, TER-119, RA3-6B2	BD
anti-mouse CD16/32 PercP-cy5.5	2.4G2	BD
anti-mouse CD34 APC-eFluor 700	RAM34	eBioscience
anti-mouse BrdU FITC		BD
Anti-mouse Gr-1 APC	RB6-8C5	BD

Supplementary Table 3. Primer sequences for qPCR.

qPCR	Forward 5'-3'	Reverse 5'-3'
NRF1	GCACCTTTGGAGAATGTGGT	GGGTCATTTTGTCCACAGAGA
mtTFA	CCCCTCGTCTATCAGTCTTGT	CTGCTTCTGGTAGCTCCCTC
β-actin	CCTAAGGCCAACCGTGAAA	CAGCCTGGATGGCTACG

Supplementary Table 4. Primer sequences for ddPCR.

ddPCR	Forward 5'-3'	Reverse 5'-3'	Probe 5'-3'
ADSS	TCGGAAATGGAGTGG	TCCCAGCCATCTAGA	TTCCTGGATT/iXNA_G

	TAATTCA	CCTT	/TTT/iXNA_G/AAGAAG CGG
IMPDH 2	GGGATCCGGCTGAAG AAATA	TTCACTGAAGTATCG GTTCTGG	TGGGTTCTCTTGATG CCATGGACA
GMPR	ACACTGTGGGGACAT TTGAGAT	GCCAGAACTCACGGC TACAT	CATGCCATGTTTACA G
β-actin	CCTAAGGCCAACCGT GAAA	CAGCCTGGATGGCTA CG	ATGACCCAGATCATG TTTGAGACCT

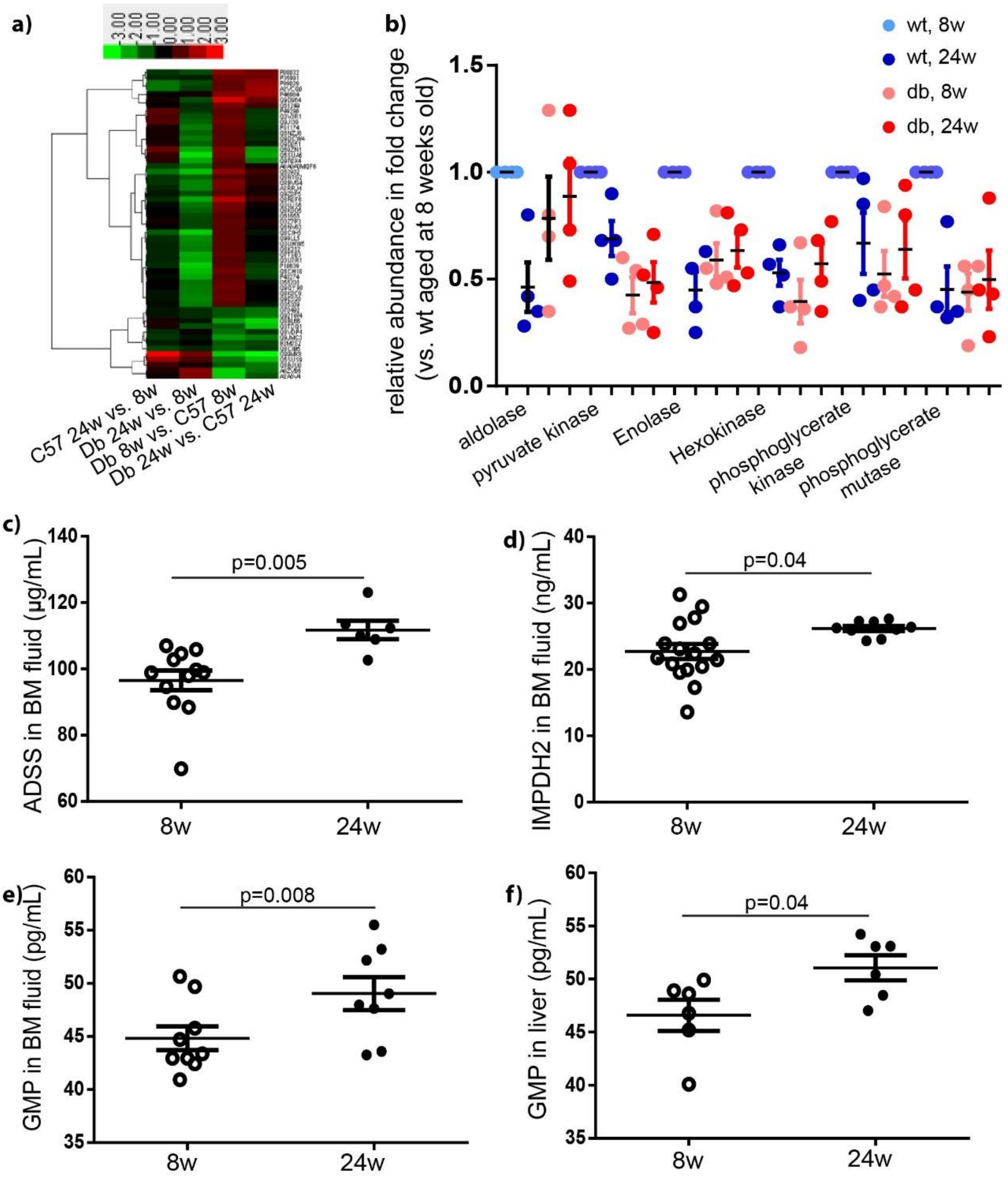

Supplementary Figure 1. Altered purine metabolism in bone marrow niche of diabetic db/db mice. Bone marrow fluid was isolated from four groups of mice (n=4 per group) and analyzed by LC-MS. **a)** Heat map among wide type and db/db mice at the age of 8 or 24 weeks old by Cluster 3.0. **b)** Glycolysis-related enzymes measured by mass spectrometry. **c)** and **d)** Validation of ADSS, IMPDH2 and GMP concentrations in bone marrow (BM) fluid extracted from db/db at 8weeks and 24 weeks old by ELISA. n=6-16. **e) and f)** Guanosine monophosphate content in liver tissue of db/db mice by ELISA assay. n=6. Data are represented as mean ± SEM. Unpaired, 2-tailed Student's t test or non-parametric Mann Whitney analysis was used to compared two groups. ADSS: adenylosuccinate synthetase. GMP: guanosine monophosphate. IMPDH2: IMP dehydrogenase 2.

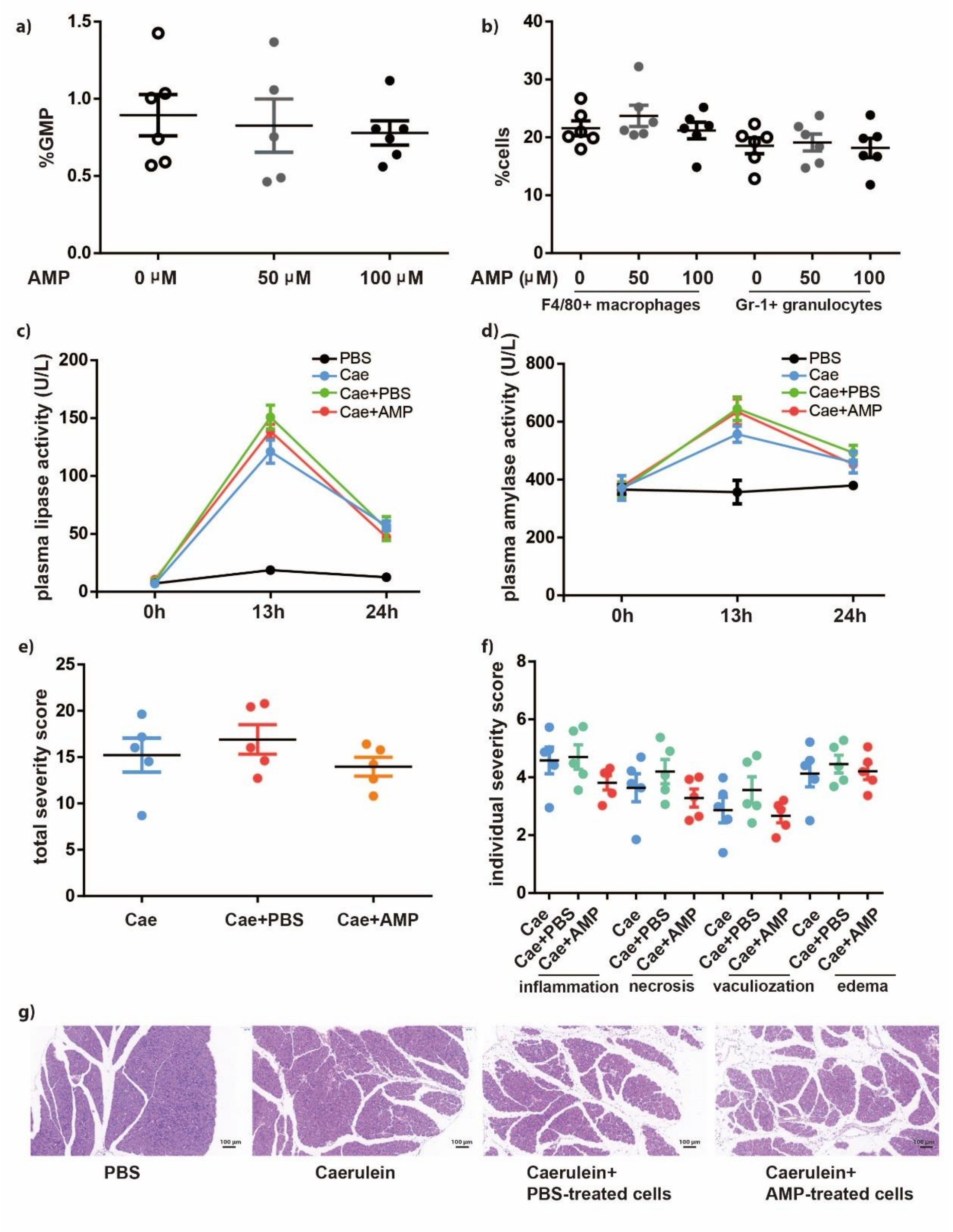

Supplementary Figure 2. Effect of adenosine monophosphate (AMP) on myeloid cell production and acute pancreatitis progression. a) and **b)** Lineage$^{-/low}$ cells of cultivated with AMP for 3 days or 5 days to assess frequency of GMP or myeloid cells by FACS analysis, respectively. n=5-6. AP model was established in wild-type recipients by ten caerulein injections with 1-hour interval. One hour after the last caerulein injection, 1x10^7 cells were injected into the recipient with established AP. **c)** and **d)** Serum lipase activity and amylase activity. **e)** and **f)** total severity score and individual severity score. n=5. **g)** representative H&E staining. Scale bar =100 μm. One-way ANOVA with Dunnett was used to compare groups against control.

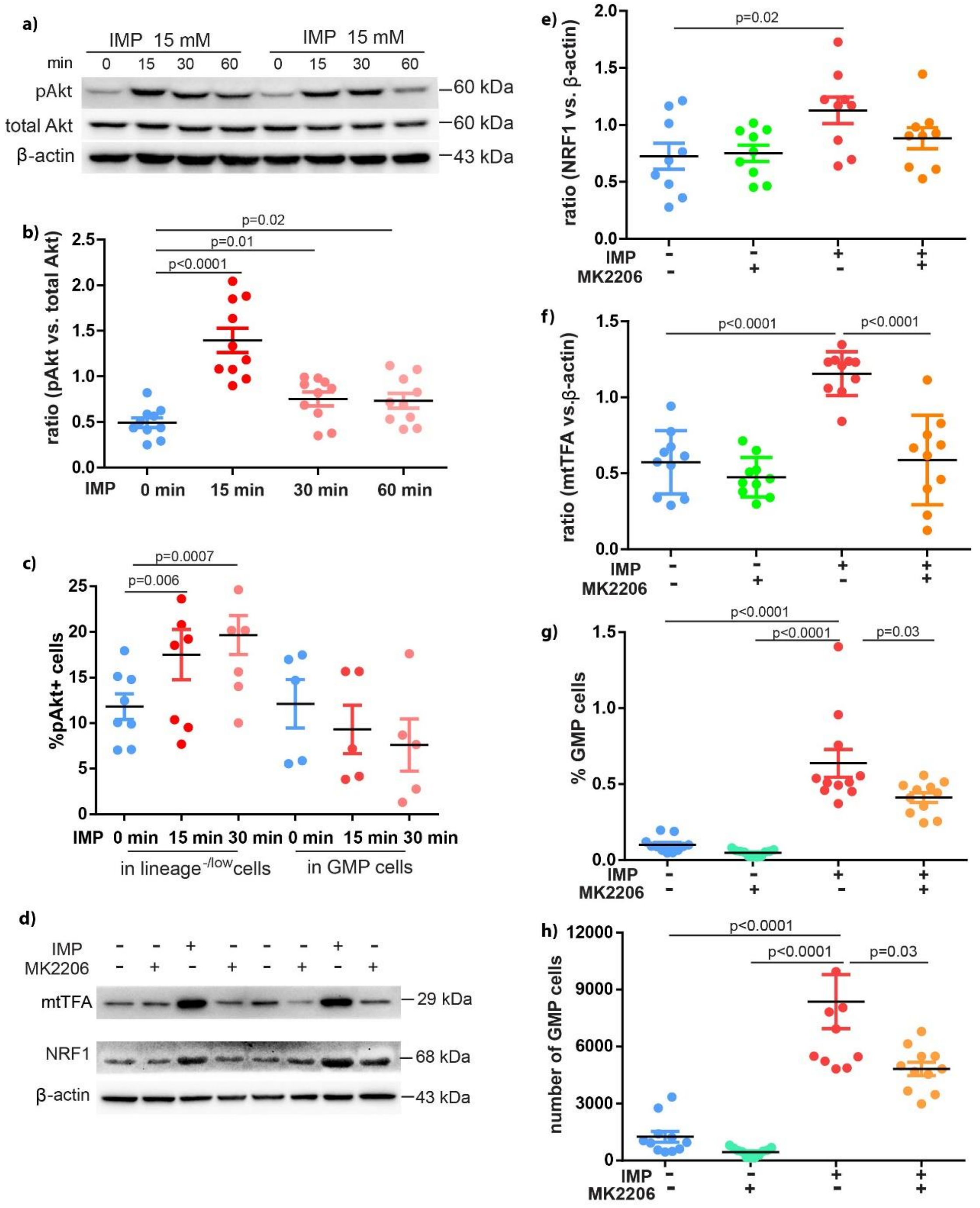

Supplementary Figure 3. Inosine monophosphate treatment induced Akt activation in lineage$^{-/low}$ cells. a) and **b)** Western blot of phosphorylation of Akt, total Akt and β-actin in lineage$^{-/low}$ cells after IMP exposure. Equal amounts of total protein lysates were immunoblotted with β-actin antibody, n=10. **c)** Frequency of pAkt+ cells in lineage$^{-/low}$ cells or GMP cells stimulated with IMP, n=9. **d) - f)** western blot of mtTFA, NRF1, and β-actin expression in lineage$^{-/low}$ cells exposed to IMP in the presence or absence of MK2206 for 72 hours, n=10. **g)** and **h)** Frequency and absolute number of GMP cells in lineage$^{-/low}$ cells treated with IMP in the presence or absence of MK2206 for 72 hours, n=9. One-way ANOVA with Dunnett was used to compare groups against control, followed by Unpaired, 2-tailed Student's t test to compare the difference

between each treated group and non-treated ones. IMP, inosine monophosphate; GMP, granulocyte-monocyte progenitors; NRF1, Nuclear respiratory factor 1; mtTFA, mitochondrial transcription factor A; MK2206, pAkt inhibitor.

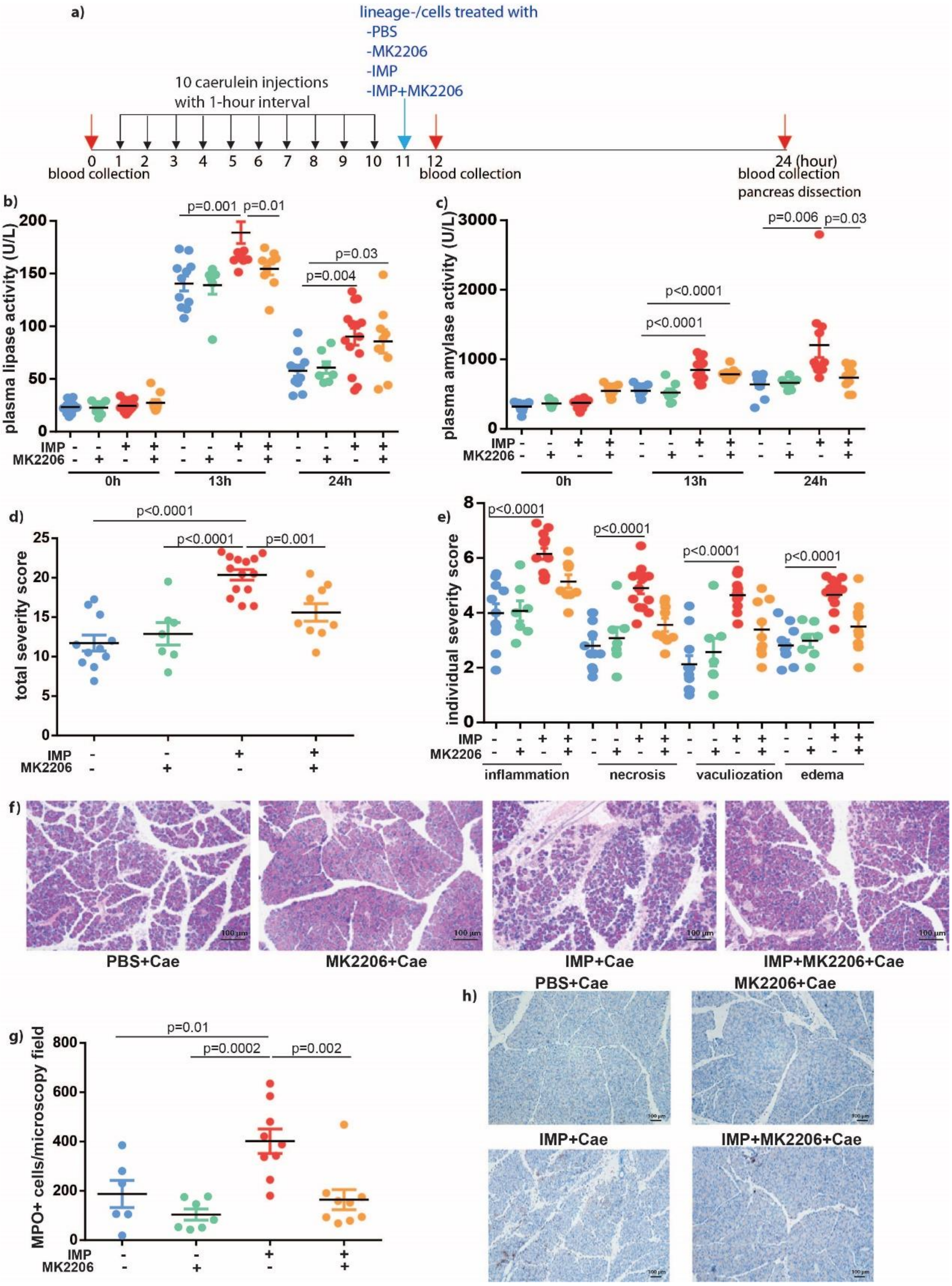

Supplementary Figure 4. Injection of inosine monophosphate-treated bone marrow cells accelerated acute pancreatitis via Akt activation. **a)** Experimental design. Lineage$^{-/low}$ cells of cultivated with IMP in the presence or absence of MK2206 for 7 days. AP model was established in wild-type recipients by ten caerulein injections with 1-hour interval. One hour after the last caerulein injection, 1x10^{7} cells were injected into the recipient with established AP. **b)** and **c)** Serum lipase activity and serum amylase activity in mice with induced AP following lineage$^{-/low}$ cells injection. **d)-**

f) The severity was assessed on H&E-stained sections. Total severity score was the sum of inflammation, necrosis, vacuolization, and edema, scale bar = 100 μm. The degree of severity was scored from 1-10, n=11-14. **g)** The number of MPO+ cells per microscopy field was numerated by ImageJ. **h)** Representative pictures of MPO-staining, scale bar = 100 μm, n=11-14. One-way ANOVA with Dunnett was used to compare groups against control. Unpaired, 2-tailed Student's t test or non-parametric Mann Whitney analysis was used to compared two groups. AP, acute pancreatitis; wt, wild-type; MPO, myeloperoxidase.

Supplementary Figure 5. Genotype of ADSS$^{+/-}$ mice.

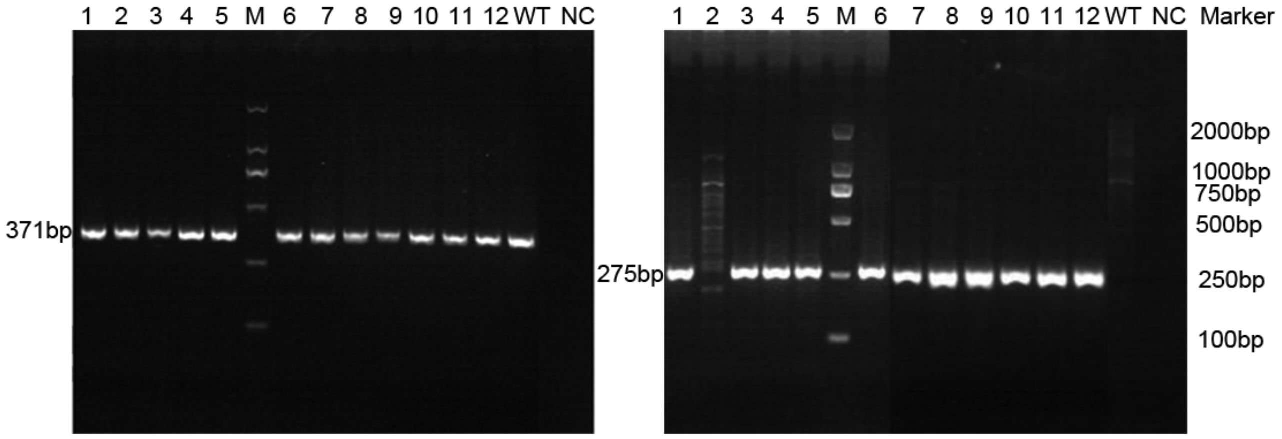

The band at 371bp or 275 bp indicated wild type or knockout of ADSS gene. ADSS, adenylosuccinate synthetase.

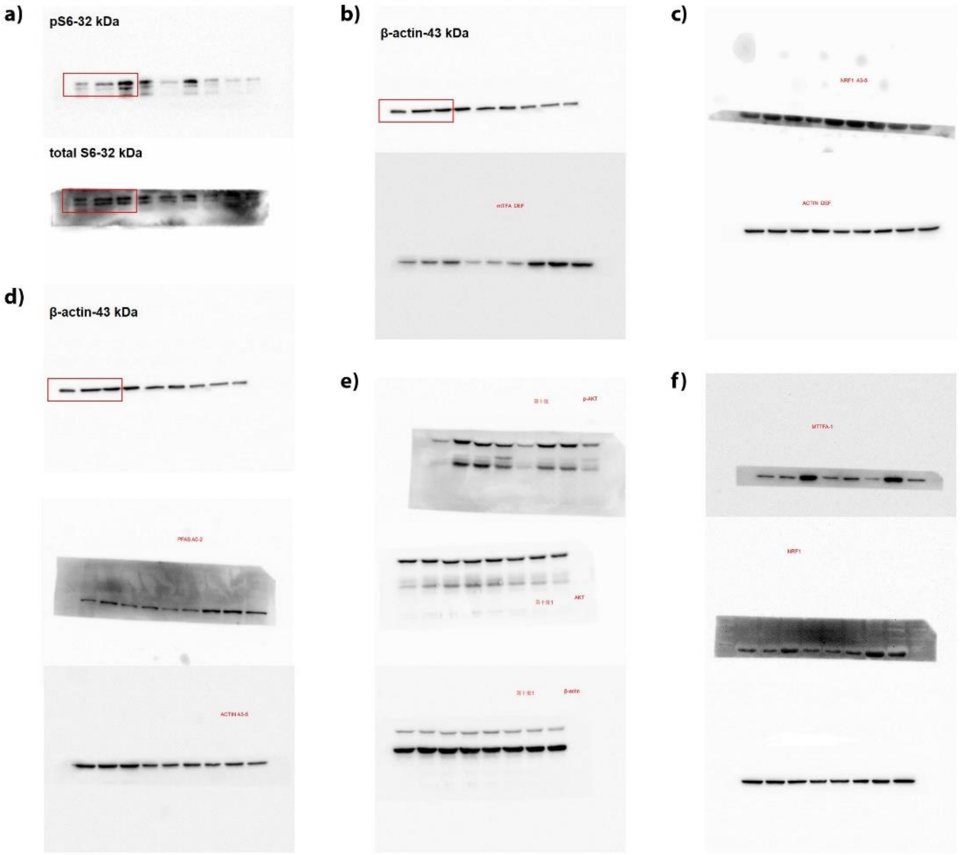

Supplementary Figure 6. Uncropped and unedited blot/gel images. a) pS6, total S6 and β-actin for Fig.4 j. **b)** mtTFA and β-actin for Fig.5 b. **c)** NRF1 and β-actin for Fig.5 b. **d)** PFAS and β-actin for Fig.5 b. **e)** pAkt, total Akt and β-actin for Fig.S3a. **f)** mtTFA, NRF1 and β-actin for Fig.S3d.
