# Supplementary material for: The purine metabolite inosine monophosphate accelerates myelopoiesis and acute pancreatitis progression
Source: Commun Biol. 2022 Oct 12;5:1088. doi: 10.1038/s42003-022-04041-0 (PMC9556615; doi:10.1038/s42003-022-04041-0)
Supplement: Supplementary file 5 — Reporting Summary [file 42003_2022_4041_MOESM5_ESM.pdf]

nature portfolio

Corresponding author(s): COMMSBIO-22-0768A

Last updated by author(s): Sep 15, 2022

Reporting Summary

Nature Portfolio wishes to improve the reproducibility of the work that we publish. This form provides structure for consistency and transparency in reporting. For further information on Nature Portfolio policies, see our Editorial Policies and the Editorial Policy Checklist.

Statistics

For all statistical analyses, confirm that the following items are present in the figure legend, table legend, main text, or Methods section.

n/a	Confirmed	
☐	☒	The exact sample size (*n*) for each experimental group/condition, given as a discrete number and unit of measurement
☐	☒	A statement on whether measurements were taken from distinct samples or whether the same sample was measured repeatedly
☐	☒	The statistical test(s) used AND whether they are one- or two-sided<br>*Only common tests should be described solely by name; describe more complex techniques in the Methods section.*
☐	☒	A description of all covariates tested
☐	☒	A description of any assumptions or corrections, such as tests of normality and adjustment for multiple comparisons
☐	☒	A full description of the statistical parameters including central tendency (e.g. means) or other basic estimates (e.g. regression coefficient) AND variation (e.g. standard deviation) or associated estimates of uncertainty (e.g. confidence intervals)
☐	☒	For null hypothesis testing, the test statistic (e.g. *F*, *t*, *r*) with confidence intervals, effect sizes, degrees of freedom and *P* value noted<br>*Give P values as exact values whenever suitable.*
☐	☒	For Bayesian analysis, information on the choice of priors and Markov chain Monte Carlo settings
☐	☒	For hierarchical and complex designs, identification of the appropriate level for tests and full reporting of outcomes
☐	☒	Estimates of effect sizes (e.g. Cohen's *d*, Pearson's *r*), indicating how they were calculated

Our web collection on statistics for biologists contains articles on many of the points above.

Software and code

Policy information about availability of computer code

Data collection	No software was used.
Data analysis	No software was used.

For manuscripts utilizing custom algorithms or software that are central to the research but not yet described in published literature, software must be made available to editors and reviewers. We strongly encourage code deposition in a community repository (e.g. GitHub). See the Nature Portfolio guidelines for submitting code & software for further information.

Data

Policy information about availability of data

All manuscripts must include a data availability statement. This statement should provide the following information, where applicable:

- Accession codes, unique identifiers, or web links for publicly available datasets
- A description of any restrictions on data availability
- For clinical datasets or third party data, please ensure that the statement adheres to our policy

Data are available after publication.

Human research participants

Policy information about studies involving human research participants and Sex and Gender in Research.

Reporting on sex and gender	Sex and gender were not considered in this study.
Population characteristics	Patients with diabetes mellitus and patients with both diabetes mellitus and acute pancreatitis.
Recruitment	Inpatients who had signed Informed Consent.
Ethics oversight	Ethics Committee of Beijing Youan Hospital.

Note that full information on the approval of the study protocol must also be provided in the manuscript.

Field-specific reporting

Please select the one below that is the best fit for your research. If you are not sure, read the appropriate sections before making your selection.

☒ Life sciences ☐ Behavioural & social sciences ☐ Ecological, evolutionary & environmental sciences

For a reference copy of the document with all sections, see nature.com/documents/nr-reporting-summary-flat.pdf

Life sciences study design

All studies must disclose on these points even when the disclosure is negative.

Sample size	Male WT C57BL/6J mice at 8 weeks old and obese db/db mice at 8 and 24 weeks old were used in the study. ADSS knockout is lethal; therefore, heterozygous mice were established spCas91.1 technology (ViewSolid Biotech, Beijing, China).
Data exclusions	No data were excluded in the analysis.
Replication	All attempts at replication were successful.
Randomization	Allocation was random.
Blinding	The investigators were blinded to group allocation during data collection and analysis.

Reporting for specific materials, systems and methods

We require information from authors about some types of materials, experimental systems and methods used in many studies. Here, indicate whether each material, system or method listed is relevant to your study. If you are not sure if a list item applies to your research, read the appropriate section before selecting a response.

Materials & experimental systems

n/a	Involved in the study	
☐	☒	Antibodies
☐	☒	Eukaryotic cell lines
☒	☐	Palaeontology and archaeology
☐	☒	Animals and other organisms
☐	☒	Clinical data
☒	☐	Dual use research of concern

Methods

n/a	Involved in the study	
☒	☐	ChIP-seq
☐	☒	Flow cytometry
☒	☐	MRI-based neuroimaging

Antibodies

Antibodies used	Akt (cat. 9272, diluted 1:1000, CST, Danvers, MA, USA); phospho-Akt (Ser473) (D9E) (cat. 4060, diluted 1:1000, CST); S6 Ribosomal Protein (54D2) (cat. 2317, diluted 1:1000, CST); phospho-S6 Ribosomal Protein (Ser235/236) (cat. 2211, diluted 1:1000, CST); NRF1 (D9K6P) (cat. 46743, diluted 1:1000, CST); mtTFA (cat. ab272885, diluted 1:1000, Abcam, Cambridge, UK); PGC-1α (cat. 66369-1-Ig, diluted 1:1000, Proteintech, Chicago, IL, USA); β-actin (13E5) (cat. 4970, diluted 1:2000, CST). secondary antibodies (goat anti-rabbit IgG-HRP (cat. ZB-2301, diluted 1:5000, ZSGB-Bio, Beijing, China) and goat anti-mouse IgG-HRP (cat. ZB-2305, diluted 1:5000, ZSGB-Bio, Beijing, China)) conjugated to horseradish peroxidase. Anti-mouse Sca-1 PerCP-Cy5.5, FITC D7 (eBioscience), anti-mouse Sca-1 PE-cy7 D7 (BD), anti-mouse cKit PE 2B8 (eBioscience), anti-mouse cKit APC-H7 2B8 (BD), anti-mouse lineage cocktail APC M1/70,

	145-2C11, RB6-8C5, TER-119, RA3-6B2 (BD), anti-mouse CD16/32 PercP-cy5.5 2.4G2 (BD), anti-mouse CD34 APC-eFluor 700 RAM34 (eBioscience), anti-mouse BrdU FITC (BD)<br>Anti-mouse Gr-1 APC RB6-8C5 BD
Validation	Data was provided in the manuscript.

Eukaryotic cell lines

Policy information about cell lines and Sex and Gender in Research

Cell line source(s)	Bone marrow cells from C57/BL mice.
Authentication	Bone marrow cells were fresh cells extracted from mice and used in experiments immediately, so cells were no need to be authenticated.
Mycoplasma contamination	All cells tested negative for mycoplasma contamination.
Commonly misidentified lines (See ICLAC register)	Fresh bone marrow cells are not applicable.

Animals and other research organisms

Policy information about studies involving animals; ARRIVE guidelines recommended for reporting animal research, and Sex and Gender in Research

Laboratory animals	Male WT C57BL/6J mice at 8 weeks old and obese db/db mice at 8 and 24 weeks old were used in the study. ADSS knockout is lethal; therefore, heterozygous mice were established spCas91.1 technology (ViewSolid Biotech, Beijing, China).
Wild animals	The study did not involve in wild animals.
Reporting on sex	Male mice.
Field-collected samples	The feeding environment requires a temperature of 18-29°C, daily temperature difference ≤3°C, relative humidity of 40%-70%, fresh air ventilation times 10 times/h, airflow velocity ≤0.18m/s, differential pressure 25Pa, cleanliness 1. Ten thousand grade, ammonia concentration 15mg/m³, noise ≤60dB, illumination 150〜300Lux.
Ethics oversight	The study is approved by Animal Ethics Committee of Capital Medical University.

Note that full information on the approval of the study protocol must also be provided in the manuscript.

Clinical data

Policy information about clinical studies

All manuscripts should comply with the ICMJE guidelines for publication of clinical research and a completed CONSORT checklist must be included with all submissions.

Clinical trial registration	LL-2021-133-K (Ethics Committee of Beijing Youan Hospital).
Study protocol	Type 2 diabetic patients with or without AP were enrolled in the Department of Cardiology of Yangzhou hospital, the Department of Endocrinology of Beijing TongRen hospital, and the Department of Science and Technology of Beijing Youan hospital from March 2016 to May 2017.
Data collection	Diabetes was defined as either fasting plasma glucose levels ≥ 7.0 mmol/L or glucose levels ≥11.0 mmol/L at 2 hours after an orally administered glucose load of 75 g. Hypertension was defined as blood pressure of ≥ 140 mmHg systolic, 90 mmHg diastolic, or the use of antihypertensive drugs. Additional characteristics including age, body weight, height, systolic blood pressure, diastolic blood pressure, heart rate, disease history and medications were recorded.
Outcomes	Not applicable.

Flow Cytometry

Plots

Confirm that:

☒ The axis labels state the marker and fluorochrome used (e.g. CD4-FITC).

☒ The axis scales are clearly visible. Include numbers along axes only for bottom left plot of group (a 'group' is an analysis of identical markers).

☒ All plots are contour plots with outliers or pseudocolor plots.

☒ A numerical value for number of cells or percentage (with statistics) is provided.

Methodology

Sample preparation	Lineage-/low cells were isolated from WT BMCs using the lineage cell depletion kit (Miltenyi Biotech, BergischGladbach, Germany), in accordance with the manufacturer's instructions. After isolation, lineage-/low cells were seeded at a density of 5 × 105 cells per well in low adsorption 24-well plates. Cells were cultured in SFEM medium (STEMCELL Technologies, Vancouver, Canada) supplemented with 10 ng/mL stem cell factor (R&D, USA), 10 ng/mL thrombopoietin (R&D, USA) and 10 ng/mL IL-3 (R&D, USA). During the study, the cells were exposed to either PBS or 15 µM IMP (MLBio, Shanghai, China) for 24 or 72 hours, respectively. After harvesting, the cells were counted and stained with GMP surface markers before being subjected to analysis by FACS. For inhibitor experiments, MK2206 (MLBio, Shanghai, China) was added at a concentration of 5 mM for 1 hour before the addition of IMP.
Instrument	BDIS
Software	Flowjo
Cell population abundance	Leukocytes, lymphocytes, monocytes, granulocytes, red blood cells, and blood platelets in PBS were quantified with an Auto Hematology Analyzer (Mindray, BC-2800Vet, Shanghai, China).
Gating strategy	Multicolor analysis for hematopoietic stem/progenitor cells (HSPCs), common myeloid progenitors (CMP), granulocyte-monocyte progenitors (GMP), megakaryocyte-erythroid progenitors (MEP), BMCs, and splenocytes was performed by FACS as previously described (described in manuscript).

☒ Tick this box to confirm that a figure exemplifying the gating strategy is provided in the Supplementary Information.
